# Genetic Variants in HSP70 Family and BAG Co-Chaperone Genes: Associations with Coronary Artery Disease Risk and Potential Regulatory Effects

**DOI:** 10.3390/ijms27167299

**Published:** 2026-08-15

**Authors:** Olga Polshvedkina, Ksenia Kobzeva, Yuriy L. Orlov, Olga Bushueva

**Affiliations:** 1 Laboratory of Genomic Research, Research Institute for Genetic and Molecular Epidemiology, Kursk State Medical University, 305041 Kursk, Russia; polshvedkinaolga@yandex.ru (O.P.);; 2 Department of Biology, Medical Genetics and Ecology, Kursk State Medical University, 305041 Kursk, Russia; 3 Institute of Digital Biodesign and AI in Medicine, Sechenov First Moscow State Medical University of the Russian Ministry of Health (Sechenov University), 119991 Moscow, Russia; orlov_yu_l@staff.sechenov.ru; 4 Agrarian and Technological Institute, Patrice Lumumba Peoples’ Friendship University of Russia, 117198 Moscow, Russia

**Keywords:** ischemic heart disease, heat shock proteins, HSP70, HSPA6, BAG1, BAG3, atherosclerosis, proteostasis, chaperones

## Abstract

Heat shock proteins of the HSP70 family and their BAG co-chaperones regulate responses to oxidative stress, inflammation, apoptosis, and ischemia, all central to coronary artery disease (CAD) pathogenesis. The contribution of genetic variants within HSP70-family and BAG co-chaperone genes to CAD susceptibility remains unclear. Thus, we sought to evaluate associations of HSP70- and BAG-related SNPs with CAD risk and to characterize their potential regulatory effects using comprehensive bioinformatic analyses. A case–control cohort of 834 CAD patients and 1328 controls of Russian ethnicity was genotyped for 13 SNPs. Associations with CAD susceptibility and traits were tested using log-additive regression with adaptive permutation. Loci underwent functional annotation. The C allele of BAG1 rs706121 was associated with increased CAD risk overall (OR = 1.24, p_perm_ = 0.019), in males (OR = 1.39, p_perm_ = 0.002), and in smokers (OR = 1.39, p_perm_ = 0.020). BAG3 rs196329 was associated with lower risk in males (A allele: OR = 0.82, p_perm_ = 0.040), whereas HSPA6 rs753856 was associated with reduced risk in physically active individuals (G allele: OR = 0.61, p_perm_ = 0.008). Additional associations involved clinical or biochemical traits. Functional annotation identified potential regulatory effects, including eQTL associations, overlap with histone marks, and allele-dependent changes in transcription factor binding. HSP70 and BAG variants may contribute to CAD susceptibility and support sex- and lifestyle-informed risk assessment.

## 1. Introduction

Coronary artery disease (CAD) remains one of the leading causes of morbidity and mortality worldwide and represents a major clinical and socioeconomic burden [1]. The disease is driven primarily by the development and progression of atherosclerotic lesions within the coronary arteries, which may ultimately lead to myocardial ischaemia and infarction [2]. CAD is a complex multifactorial disorder arising from the combined effects of genetic susceptibility, environmental exposures, lifestyle factors, and metabolic abnormalities, including dyslipidaemia, hypertension, obesity, smoking, physical inactivity, and impaired haemostatic regulation [3]. These factors promote endothelial dysfunction, oxidative stress, chronic inflammation, vascular remodeling, and thrombosis, all of which contribute to disease initiation and progression [4].

Heat shock proteins constitute an essential component of the cellular defense machinery and help maintain protein homeostasis under physiological and pathological stress [5,6]. Among them, members of the 70 kDa heat shock protein family (HSP70) act as ATP-dependent molecular chaperones that facilitate protein folding, prevent aggregation, promote the refolding or degradation of damaged proteins, and regulate apoptosis and cell survival [7,8]. Their activity is tightly controlled by co-chaperones, including members of the BCL2-associated athanogene family, such as BAG1 and BAG3, which modulate substrate recognition, nucleotide exchange, and the balance between protein refolding and degradation [7].

The HSP70–BAG chaperone system is particularly relevant to cardiovascular biology because endothelial cells, vascular smooth muscle cells, immune cells, and cardiomyocytes are continuously exposed to oxidative, inflammatory, metabolic, mechanical, and ischaemic stress [9,10]. Several HSP70 family members have been implicated in vascular and cardiac homeostasis. HSPA6, also known as HSP70B′, is strongly induced under severe cellular stress and has been reported to inhibit vascular smooth muscle cell proliferation [11]. HSPA8 participates in constitutive protein quality control and chaperone-mediated autophagy [12], whereas HSPA9 contributes to mitochondrial proteostasis [13]. *HSPA12B* is highly expressed in endothelial cells and has been linked to vascular integrity and angiogenic responses [14]. BAG3, in turn, is important for sarcomere maintenance, mechanotransduction, calcium homeostasis, and cardiomyocyte survival [15], while BAG1 regulates apoptosis and signaling pathways involving BCL2, RAF, and AKT [16]. Dysregulation of these genes may therefore influence endothelial function, inflammatory responses, plaque stability, myocardial adaptation, and thrombosis.

Genetic variation in *HSP70* and *BAG* genes may alter their expression, regulatory activity, or interactions with other proteins and thereby modify susceptibility to CAD and its clinical manifestations. Previous studies have linked polymorphisms in HSP-related genes to hypertension, myocardial infarction, lipid metabolism, and other cardiovascular phenotypes [17,18,19,20,21]. However, relatively little is known about whether variants across several HSP70 family members and their co-chaperones jointly contribute to CAD risk, age at disease onset, coronary stenosis, lipid profiles, and coagulation-related traits. Moreover, the effects of such variants may depend on sex, smoking status, physical activity, and other environmental or behavioral factors [22,23,24]. Investigation of these subgroup-specific associations is therefore important for understanding how genetic variation in the cellular stress-response system may interact with established CAD risk factors.

In the present study, we investigated selected single-nucleotide polymorphisms (SNPs) in genes encoding HSP70 family members, including *HSPA1A*, *HSPA4*, *HSPA6*, *HSPA8*, *HSPA9*, *HSPA12B*, and *HSPA14*, and in the co-chaperone genes *BAG1* and *BAG3*. We assessed their associations with CAD susceptibility in the overall study population and in subgroups stratified by sex, smoking status, and physical activity. We further examined their relationships with age at CAD onset, blood lipid levels, coagulation parameters, and the severity of coronary artery stenosis. Finally, we integrated the genetic associations with eQTL data, histone modification profiles, and predictions of allele-specific transcription factor binding and functional enrichment to explore potential molecular mechanisms underlying the observed effects.

## 2. Results

### 2.1. HSP70 and BAG Polymorphisms Contribute to CAD Susceptibility

The preliminary analysis included assessment of genotype distributions in CAD patients and healthy controls as well as their correspondence to the Hardy–Weinberg equilibrium (HWE) (Supplementary Table S1). Several variants showed deviations from HWE in the CAD group, including rs753856 *HSPA6*, rs1043618 *HSPA1A*, rs706121 *BAG1*, and rs1136141 *HSPA8*. Such deviations in affected individuals may reflect disease-associated enrichment of particular genotypes. The variant rs706121 *BAG1* also deviated from HWE in the control group. To exclude a possible genotyping error, rs706121 was re-genotyped, and the repeat analysis was fully concordant with the original results, making a systematic technical error less likely. Nevertheless, deviation from Hardy–Weinberg equilibrium may also result from random sampling variation, population substructure, non-random recruitment or selection bias, or biological effects associated with the locus. The variant was therefore retained in the subsequent analyses, but the corresponding findings were interpreted with caution.

To assess whether the SNPs were associated with CAD risk, we applied an additive genetic model. We performed association analysis in the entire group to assess the SNP risk independent of environmental and other risk factors (Supplementary Table S2) and in the groups stratified by sex, smoking status and levels of physical activity (Supplementary Table S3).

**Figure 1:**
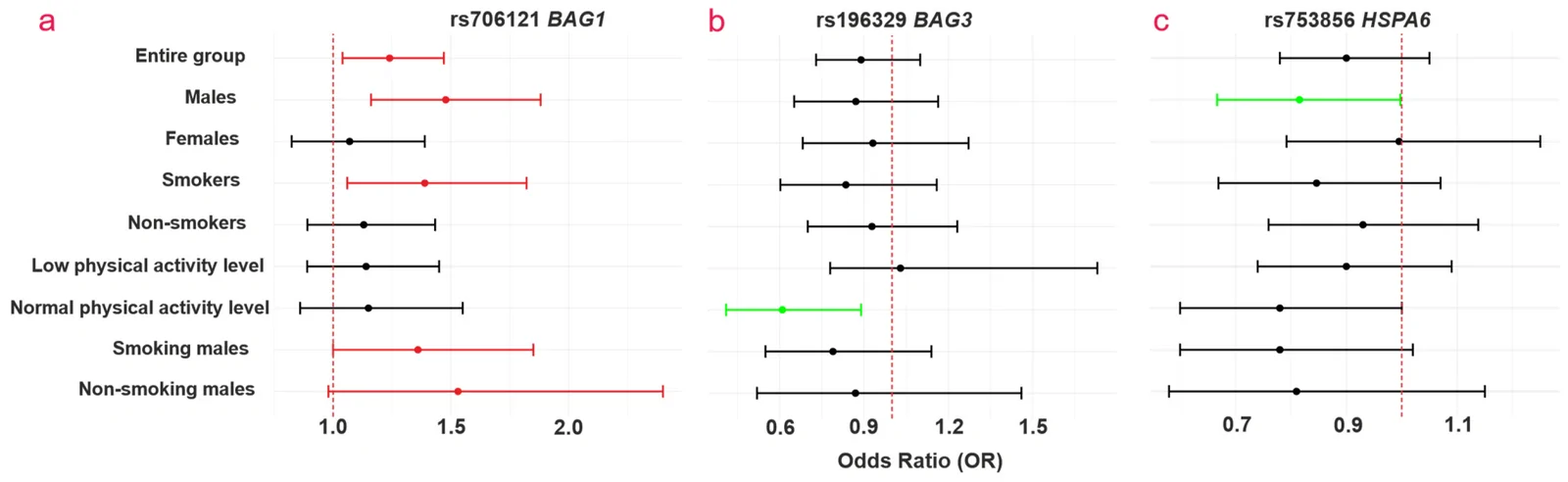
HSPs associations with CAD risk in analyzed groups. (**a**) Forest plot for rs706121 *BAG1* associations with CAD risk; (**b**) forest plot for rs196329 *BAG3* associations with CAD risk; (**c**) forest plot for rs753856 *HSPA6* associations with CAD risk. Note: dots represent ORs; 95% CI is represented by bars. Red and green indicate significant associations with risk and protective effects, respectively, black indicates non-significant associations.

Subsequently, allele C of SNP rs706121 *BAG1* was associated with increased risk of CAD in the entire group (OR = 1.24, 95%CI 1.04–1.47, *p*_perm_ = 0.019), in males (OR = 1.39, 95% CI 1.16–1.88, *p*_perm_ = 0.002), and in smokers (OR = 1.39, 95%CI 1.06–1.82, *p*_perm_ = 0.02) (Figure 1a). As males usually form the majority of smoking cohorts, to rule out sex or smoking effects in SNP association, we additionally stratified the male group by smoking status (Supplementary Table S4). Interestingly, it turned out that the risk effect of SNP rs706121 *BAG1* remains both in smoking (OR = 1.36, 95%CI 1.00–1.85, *p*_perm_ = 0.04) and non-smoking males (OR = 1.53, 95%CI 0.98–2.4, *p*_perm_ = 0.046) (Figure 1a), though with reduced strength of associations, which may be due to the smaller size of the analyzed groups. These results suggest that the sex effect is more significant than smoking status.

Furthermore, two additional SNPs displayed protective effects: rs196329 *BAG3* reduced CAD risk in males (effect allele A, OR = 0.82, 95% CI 0.67–1, *p*_perm_ = 0.04) (Figure 1b) and rs753856 *HSPA6* reduced CAD risk in the patient group with normal physical activity levels (effect allele G, OR = 0.61, 95% CI 0.41–0.89, *p*_perm_ = 0.008) (Figure 1c).

### 2.2. HSP70 and BAG Polymorphisms Modulate Clinical and Biochemical Parameters in CAD Patients

In the next step of K-W and M-U analysis, we were interested in assessing SNPs’ effect on clinical and biochemical parameters that are significant for CAD pathogenesis.

**Figure 2:**
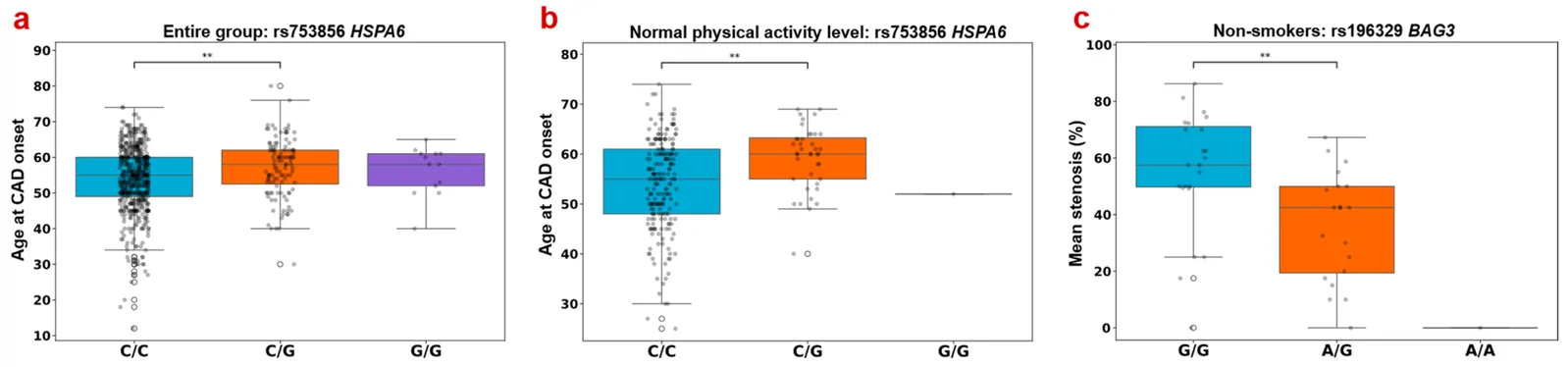
Associations of polymorphic variants in *HSP70*-family genes with age at coronary artery disease onset and coronary artery stenosis. Boxplots show age at CAD onset according to genotype for (**a**,**b**) rs753856 *HSPA6* in the overall cohort (*p_kw_* = 0.0026) and participants with sufficient physical activity (*p_kw_* = 0.0017), respectively; Boxplot (**c**) shows mean coronary artery stenosis according to genotype for (**b**) rs196329 *BAG3* (*p_kw_* = 0.0029) in non-smoking CAD patients. Boxes indicate the median and interquartile range, whiskers extend to 1.5 times the interquartile range, and individual points represent study participants. Overall differences among genotype groups were assessed using the Kruskal–Wallis test. Significant pairwise comparisons were evaluated using two-sided Mann–Whitney U tests and are indicated by asterisks (** *p* < 0.01).

Carriers of the C/G genotype rs753856 *HSPA6* had a higher age of CAD onset than normal homozygotes carrying the C/C genotype (Figure 2a,b). These associations were distinctive for the entire group (*p*_kw_ = 0.0026) and for patients with normal physical activity levels (*p*_kw_ = 0.0017). Additionally, we found that SNP rs196329 in the *BAG3* gene was associated with reduced mean coronary artery stenosis lesions in non-smoking CAD patients (*p*_kw_ = 0.0029) (Figure 2c).

### 2.3. Functional Annotation of HSP70 and BAG Polymorphisms Associated with CAD Risk

In the next step of our study, we resorted to bioinformatic resources and tools to functionally annotate the possible underlying molecular mechanisms of the risk-associated SNPs.

First, this included eQTL analysis in blood, vessels and heart tissues using data from the GTEx Portal and the eQTL gene browser (Table 1).

According to the GTEx Portal, associations with eQTL effects were found only for rs706121 *BAG1*: allele T of this SNP is linked to lower expression of the *NFX1* gene in the aorta (NES = −0.15, *p* = 7.6 × 10^−7^), the tibial artery (NES = −0.093, *p* = 1.2 × 10^−4^), the atrial appendage (NES = −0.21, *p* = 2.1 × 10^−11^), the left ventricle (NES = −0.13, *p* = 2.5 × 10^−6^), and whole blood (NES = −0.25, *p* = 1.0 × 10^−27^).

As for data on eQTL effects in whole blood from the eQTL gene browser, it was found that allele G of SNP rs753856 *HSPA6* influences the simultaneous expression of several genes: it is associated with increased expression of *RP11-5K23.3*, *FCGR2A*, and *FCGR2C*, and lowered expression of *FCGR2B*, *FCRLB*, and *RP11-122G18.5* (Table 1). Allele C of rs706121 *BAG1* is associated with heightened expression levels of *AQP3*, *NOL6*, and *NFX1*, and with diminished expression of *BAG1* itself. Similarly, allele A of rs196329 *BAG3* is associated with lowered expression of *BAG3* and higher expression of *MCMBP*.

Second, with HaploReg data, it was possible to assess histone modification marks of the risk-associated SNPs in the blood, heart, and arteries (no marks for the three SNPs in the aorta were found) (Table 2). SNP rs753856 *HSPA6* is linked to H3K4me1 marking enhancers both in blood cells and in the left ventricle, to H3K4me3 marking promoters in blood cells and the right ventricle, and, only in blood cells, to H3K9ac marking promoters. SNP rs706121 *BAG1* is associated with H3Kme1 and H3K27ac marking enhancers and H3Kme3 marking promoters only in blood cells. SNP rs196329 *BAG3* is linked to H3K4me1 marking enhancers in the heart tissues (right and left ventricles and right atrium) and to H3K27ac marking enhancers in blood cells and right and left ventricles.

Third, we conducted enrichment analysis against GO terms for transcription factors that bind exclusively to effect or reference alleles of analyzed SNPs (see the list of TFs in Supplementary Tables S5–S7).

**Figure 3:**
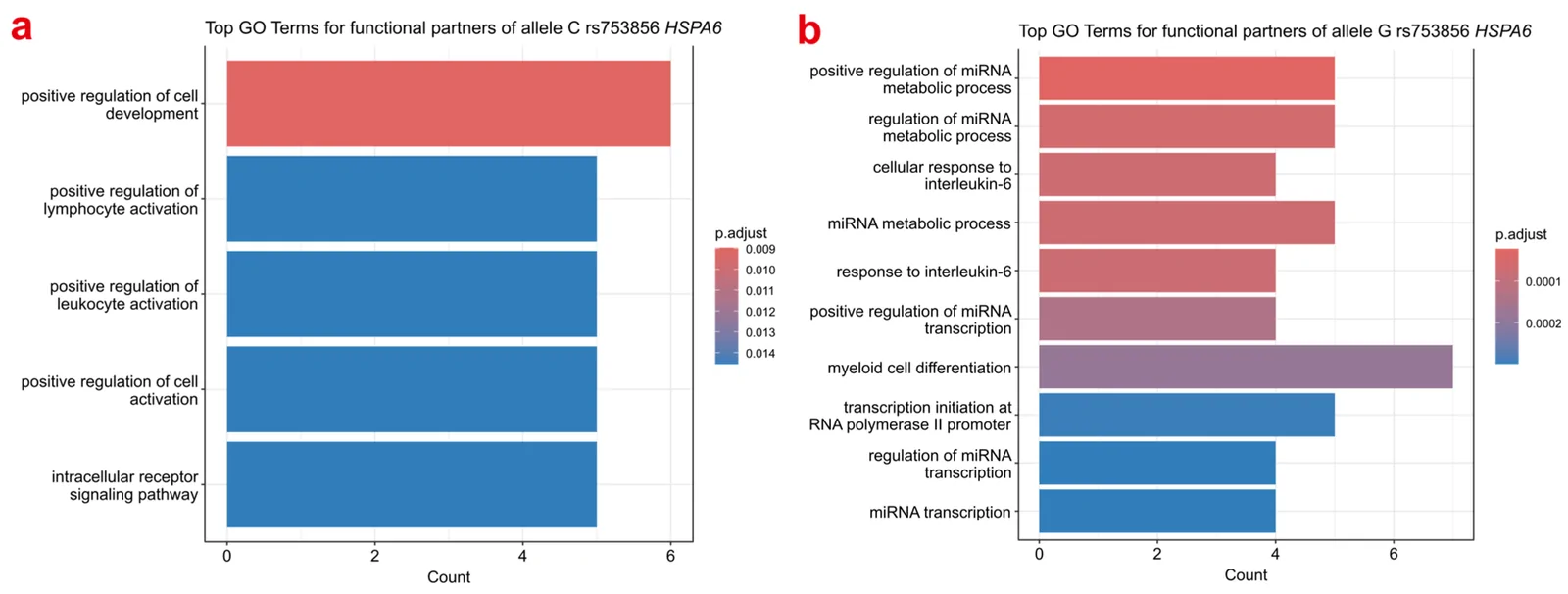
Gene Ontology enrichment of transcription factors predicted to bind allele specifically to rs753856 *HSPA6*. Gene Ontology biological process enrichment analysis was performed for transcription factors predicted to bind exclusively to either allele of rs753856. (**a**) Transcription factors preferentially binding the CAD risk-associated C allele. (**b**) Transcription factors preferentially binding the protective G allele. The complete lists of allele-specific transcription factors are provided in Supplementary Table S5.

While TFs binding to the risk allele C of rs753856 *HSPA6* are enriched in GO terms associated with cell activation, including activation of lymphocytes and leukocytes, as well as in the intracellular signaling pathway (Figure 3a), TFs binding to the protective allele G of rs753856 *HSPA6* are enriched in the following GO terms: miRNA metabolic processes, cellular response to IL-6 and myeloid cell differentiation (Figure 3b).

**Figure 4:**
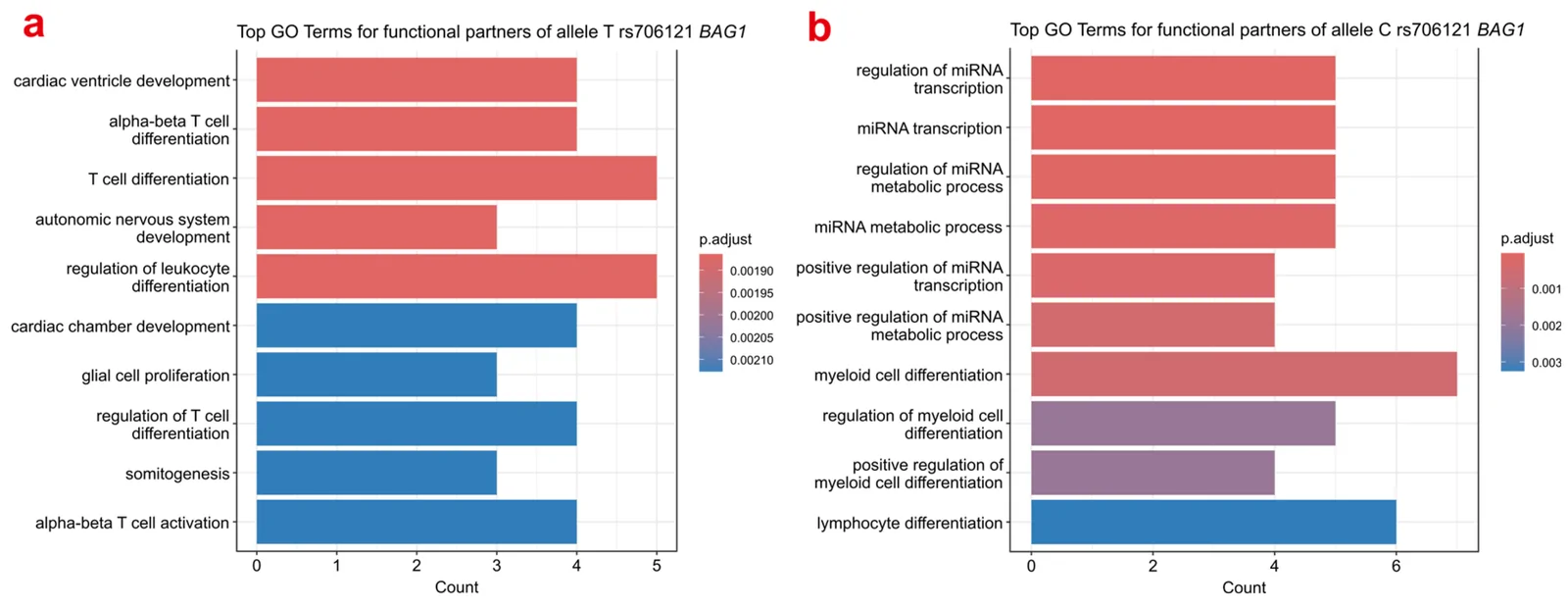
Gene Ontology enrichment of transcription factors predicted to bind allele specifically to rs706121 *BAG1*. Gene Ontology biological process enrichment analysis was performed for transcription factors predicted to bind exclusively to either allele of rs706121. (**a**) Transcription factors preferentially binding the protective T allele. (**b**) Transcription factors preferentially binding the CAD risk-associated C allele. The complete lists of allele-specific transcription factors are provided in Supplementary Table S6.

Notably, TFs for the protective allele T rs706121 *BAG1* are significantly enriched in GO terms associated with immunity and heart development, including T cell, leukocyte differentiation and cardiac ventricle and chamber development (Figure 4a). TFs binding to the risk allele C rs706121 *BAG1* are mostly enriched in terms linked to miRNA transcription and myeloid cell and lymphocyte differentiation (Figure 4b).

**Figure 5:**
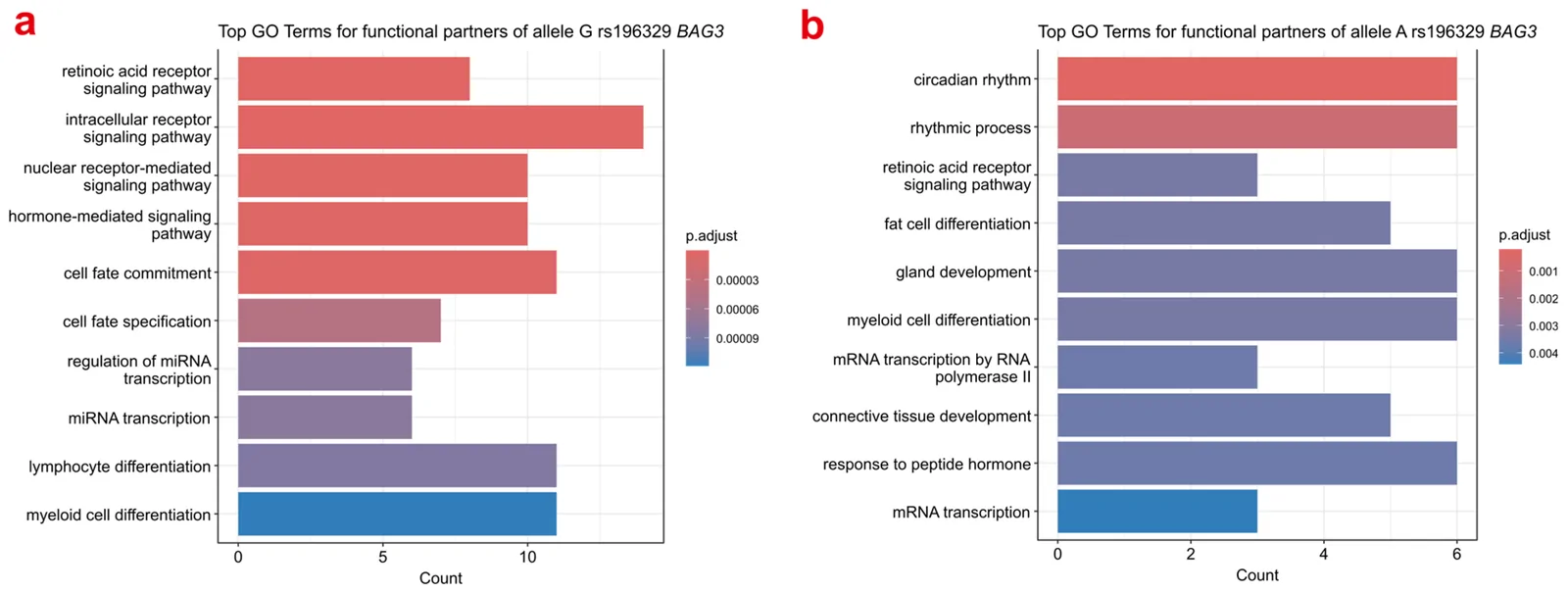
Gene Ontology enrichment of transcription factors predicted to bind allele specifically to rs196329 *BAG3*. Gene Ontology biological process enrichment analysis was performed for transcription factors predicted to bind exclusively to either allele of rs196329. (**a**) Transcription factors preferentially binding the CAD risk-associated G allele. (**b**) Transcription factors preferentially binding the protective A allele. The complete lists of allele-specific transcription factors are provided in Supplementary Table S7.

GO terms for TFs binding to the DNA site created by the risk allele G of rs196329 *BAG3* similarly include lymphocyte, myeloid cell differentiation and signaling pathways mediated by retinoic acid receptors, intracellular receptors, nuclear receptors, and hormone (Figure 5a). As for GO terms for TFs binding to the protective allele A rs196329 *BAG3*, it preserves the retinoic acid receptor signaling pathway, myeloid cell differentiation, and circadian rhythm differentiation, fat cell differentiation, response to peptide hormone, and connective tissue development (Figure 5b).

## 3. Discussion

In this study, we show that SNPs in genes encoding HSP70 family members and their co-chaperones (BAG1 and BAG3) are associated with CAD risk, clinical phenotypes, and biochemical parameters. Specifically, rs706121 *BAG1* was associated with increased CAD risk in the entire group, males and smokers, while rs196329 *BAG3* was linked to CAD protection in males, and rs753856 *HSPA6* was associated with lower CAD risk in a cohort with normal physical activity levels. Beyond risk modulation, the SNP rs753856 *HSPA6* influenced CAD onset age and the SNP rs196329 *BAG3*—coronary artery stenosis severity. Importantly, none of the variants associated with CAD in our cohort have previously been established as a CAD susceptibility locus in large-scale genome-wide association studies. Bioinformatic analyses further revealed potential molecular mechanisms, including eQTL effects, histone modifications, and differential TF binding, which may underlie these associations. These findings underscore the role of HSP70 and co-chaperones in CAD pathophysiology, potentially through stress response, inflammation, and vascular homeostasis pathways.

In total, we identified three key risk-modulating loci: rs753856 in *HSPA6* (Heat Shock Protein Family A (HSP70) Member 6), rs706121 in *BAG1* (BCL2-associated athanogene 1), and rs196329 in *BAG3* (BCL2-associated athanogene 3). These variants are located in genes integral to cellular stress responses, protein folding, and anti-apoptotic processes, which are perturbed in CAD due to oxidative stress, inflammation, and endothelial dysfunction [25,26].

The protective effect of rs753856 (G allele) in *HSPA6* was observed specifically in those with normal physical activity, suggesting an interaction with lifestyle factors. HSPA6, also known as HSP70B’, is inducible and expressed under stress and has been implicated in suppressing vascular smooth muscle cell (VSMC) proliferation [11]. Further eQTL analysis showed that the G allele of rs753856 associates with altered expression of immune-related genes (e.g., increased *FCGR2A*/*FCGR2C* and decreased *FCGR2B*/*FCRLB*), potentially modulating Fc gamma receptor-mediated inflammation in atherosclerosis [27,28], while TF enrichment for the G allele points to miRNA metabolism, IL-6 response, and myeloid differentiation—pathways relevant to CAD’s inflammatory milieu [29,30]. Clinically, the G allele correlated with delayed CAD onset (in the entire and normal activity groups).

For the C allele of rs706121 in *BAG1*, we observed an association with increased CAD risk, particularly in males and smokers, independent of smoking status upon stratification. BAG1 interacts with Raf kinases, Akt, and Bcl-2, influencing cell survival and proliferation [16]. QTL data revealed the rs706121 C allele’s association with reduced *BAG1* expression but increased *AQP3*, *NOL6*, and *NFX1*—genes tied to aquaporin function, nucleolar processes, and transcription regulation, potentially disrupting cellular homeostasis. TF analysis showed that the C allele was enriched in miRNA transcription and myeloid/lymphocyte differentiation, while the protective T allele was enriched in immunity-and cardiac-development terms (e.g., T-cell differentiation, ventricle/chamber development).

The protective A allele of rs196329 in *BAG3* was male-specific, consistent with several studies reporting sex-specific effects for this gene in diseases [31,32]. BAG3 maintains sarcomere integrity via interactions with Hsc70, CapZb1, and actin, regulates contractility/calcium homeostasis [15,33], and responds to stressors like proteasome inhibition or oxidants [34]. In CAD contexts, BAG3 has vasorelaxation properties, reducing blood pressure [10]. Our findings link the rs196329 *BAG3* SNP to reduced stenosis lesions only in non-smokers. eQTL effects showed that the A allele of rs196329 was associated with decreasing *BAG3* but increasing *MCMBP* (minichromosome maintenance binding protein) expression levels, potentially affecting DNA replication and cell cycle in vascular cells [35]. TF enrichment for the A allele included fat cell differentiation, peptide hormone response, and connective tissue development—relevant to CAD’s metabolic and fibrotic aspects—while the G allele emphasized retinoic acid/nuclear receptor signaling and immune differentiation.

In conclusion, variants in genes encoding HSP70 members and BAG co-chaperones were found to modulate CAD risk and related phenotypes, with implications for personalized medicine (e.g., sex/smoking-stratified risk assessment). Future studies should validate these in diverse populations, explore causal mechanisms via in vitro models, and assess therapeutic targeting (e.g., HSP70 inducers) to mitigate CAD burden.

Several limitations should be acknowledged. First, although the total cohort included 834 patients with CAD and 1328 controls, the statistical power was reduced after stratification by sex, smoking status, physical activity, and other lifestyle-related factors. Second, the study did not include an independent replication cohort, and the population-specific nature of the sample may limit the generalizability of the results to other ancestries. Third, the candidate-gene design examined only a limited number of preselected variants and cannot capture the broader polygenic architecture of CAD. Fourth, the functional interpretations are based on publicly available eQTL data and computational predictions of chromatin features and transcription-factor binding. No experimental assays were performed to confirm allele-specific effects on gene expression, protein function, inflammation, coagulation, or vascular remodeling.

## 4. Materials and Methods

### 4.1. Study Participants

The study included 2162 unrelated individuals of Russian ethnicity from Central Russia, comprising 834 patients with CAD and 1328 healthy controls. The baseline and clinical characteristics of the participants are summarized in Supplementary Table S8. All participants or their legal representatives gave signed informed consent before enrolling in the study.

The patients were recruited from Kursk Regional Clinical Hospital and Kursk Emergency Hospital, where they were examined by qualified cardiologists. The diagnosis of CAD was established based on objective examination of patients and the results of instrumental research methods (electrocardiogram, coronary angiography) [36].

Exclusion criteria were applied: chronic liver, kidney, endocrine, or oncological diseases; cardiomyopathy; chronic obstructive pulmonary disease; collagenosis; acute and chronic infectious and inflammatory diseases; alcoholism.

The control group consisted of healthy volunteers, whose characterization has been described in detail in our previous studies [36,37].

### 4.2. Genetic Analysis

Genotyping was conducted by the Laboratory of Genomic Research at the Research Institute for Genetic and Molecular Epidemiology of Kursk State Medical University (Kursk, Russia).

Genomic DNA was extracted from thawed blood samples, and its concentration and purity were assessed using a NanoDrop spectrophotometer (Thermo Fisher Scientific, Waltham, MA, USA). The following variants were genotyped: rs1043618 in *HSPA1A*, rs753856 in *HSPA6*, rs13161158 in *HSPA4*, rs1461496, rs10892958, and rs1136141 in *HSPA8*, rs1042665 in *HSPA9*, rs862832 and rs910652 in *HSPA12B*, rs17155992 in *HSPA14*, rs706121 in *BAG1*, and rs196336 and rs196329 in *BAG3*. Candidate variants were selected based on a minor allele frequency greater than 0.05 and the technical feasibility of designing reliable genotyping probes. Moreover, to assess the potential regulatory relevance of the selected variants, we compiled publicly available functional annotations from HaploReg v4.2 [38], RegulomeDB [39], and atSNP [40] (Supplementary Table S9). European allele frequencies and HaploReg annotations were used to summarize the overlap of each SNP with promoter- and enhancer-associated histone marks, DNase-hypersensitive regions, experimentally bound proteins, altered transcription-factor motifs, QTL associations, and dbSNP functional annotations. RegulomeDB probability scores and regulatory rankings were used to estimate the likelihood that each variant has a functional regulatory effect. In addition, atSNP was used to predict whether the reference and alternative alleles differentially affect transcription-factor binding affinity.

Genotyping was performed by allele-specific probe-based real-time PCR according to our protocols. Primer and probe sequences, assay-specific annealing temperatures, and MgCl_2_ concentrations are provided in Supplementary Table S10. PCR amplification was carried out in a total reaction volume of 25 µL containing approximately 10 ng of genomic DNA, 1.5 U of Hot Start Taq DNA polymerase (Biolabmix, Novosibirsk, Russia), 0.25 of µM of each primer, 0.10 µM of each allele-specific probe, 250 µM of each dNTP, the assay-specific concentration of MgCl_2_, and 1× PCR buffer comprising 67 mM Tris-HCl (pH 8.8), 16.6 mM (NH_4_)_2_SO_4_, and 0.01% Tween-20. The thermal cycling program consisted of an initial denaturation at 95 °C for 10 min, followed by 39 cycles of denaturation at 92 °C for 30 s and annealing at the assay-specific temperature for 1 min.

To ensure quality control, 10% of the DNA samples were genotyped twice, with the case–control status blinded. The results showed over 99% concordance between the repeated tests.

### 4.3. Bioinformatics and Statistical Analysis

We used PLINK v1.9.0-b.7.7 (www.cog-genomics.org/plink/1.9/, accessed on 11 August 2026) for the statistical analysis, including Hardy–Weinberg equilibrium (HWE) and genotype frequencies assessment, association testing between genotypes and CAD risk, and genotypic effects on quantitative traits [41]. Normalization of quantitative traits was performed using R software (version 3.6.3, R Foundation for Statistical Computing, Vienna, Austria).

In the analysis of genotype association, the log-additive model was employed. Covariates, which comprised factors indicating variations in the overall biological characteristics of the studied groups (sex, age, BMI, and smoking status), were taken into account when adjusting associations in the entire group of patients and controls. Correction for multiple comparisons was performed using the adaptive permutation test (*p*_perm_). The statistically significant level was taken as *p*_perm_ < 0.05.

Associations between genotypes and quantitative clinical or biochemical traits were evaluated using non-parametric tests implemented in Python (version 3.12). For each SNP–phenotype combination, individuals with missing genotype or phenotype data were excluded. For each phenotype, differences among genotype groups were first assessed using the Kruskal–Wallis test for all investigated SNPs. The resulting Kruskal–Wallis *p*-values were adjusted across SNPs separately for each phenotype using the Benjamini–Hochberg false discovery rate procedure. Pairwise two-sided Mann–Whitney U tests were performed only for SNP–phenotype associations that remained significant after FDR correction. The *p*-values from all pairwise genotype comparisons within each SNP–phenotype combination were subsequently adjusted using the Holm method. Adjusted *p*-values below 0.05 were considered statistically significant. Analyses were performed either in the entire cohort or in predefined subgroups. Phenotype distributions across genotype groups were visualized using boxplots with jittered individual data points, and significant pairwise comparisons based on Holm-adjusted *p*-values were indicated on the plots. Data processing and statistical analyses were performed using the pandas, SciPy, and statsmodels packages, and figures were generated using Matplotlib (version 3.9.2), Seaborn (version 0.13.2), and statannotations (version 0.7.2, https://pypi.org/project/statannotations/, accessed on 11 August 2026).

Utilizing the following tools (described in detail here [42,43]), functional implications of the studied SNPs were examined:

- Because regulatory variants may influence the expression of other nearby genes through cis-regulatory mechanisms, the GTEx Portal tool was used to analyze the expression quantitative trait loci (eQTLs) effects of the studied SNPs in the blood vessels and the heart [44]. For each variant, available cis-eQTL associations were examined, and the direction of the effect of the alternative allele on gene expression was recorded.
- HaploReg (v4.2), a bioinformatics tool available at https://pubs.broadinstitute.org/mammals/haploreg/haploreg.php (accessed on 2 June 2025), was employed to assess histone modifications associated with *HSP70* SNPs in heart tissues. Histone H3 protein lysine residues at positions 27 and 9 (H3K27ac and H3K9ac, respectively), as well as mono- and tri-methylation at position 4 (H3K4me1) and position 4 (H3K4me3), were among the alterations that were examined [38].
- atSNP Function Prediction http://atsnp.biostat.wisc.edu/search (accessed on 2 June 2025): This web-based tool was used to predict whether the reference and alternative alleles of the investigated SNPs differentially affect transcription-factor (TF) binding. atSNP compares the predicted affinity of each allele for known transcription-factor binding motifs and estimates the statistical significance of allele-specific differences in binding. TFs showing evidence of altered binding affinity between the two alleles were retained for further functional interpretation [40].
- The R package clusterProfiler was used for over-representation and enrichment analysis of GO terms for TFs linked to the reference or SNP alleles [45].

## Figures and Tables

**Table 1:** Tissue-specific cis-eQTL effects of CAD-associated variants in *HSP70*-family and *BAG* co-chaperone genes.

SNP	Allele Assessed	Symbol	Z-Score	FDR
rs753856 *HSPA6* (C/**G**)	G	*RP11-5K23.3*	26.74	0
		*FCGR2B*	−15.46	0
		*FCGR2A*	6.23	0
		*FCRLB*	−5.98	1.96 × 10^−5^
		*RP11-122G18.5*	−4.64	0.009
		*FCGR2C*	4.39	0.029
rs706121 *BAG1* (T/**C**)	C	*AQP3*	10.92	0
		*NOL6*	8.32	0
		*NFX1*	7.96	0
		*BAG1*	−7.12	0
rs196329 *BAG3* (G/**A**)	A	*BAG3*	−11.89	0
		*MCMBP*	11.85	0

Note: alleles associated with CAD risk are marked in bold.

**Table 2:** Histone modification annotations of CAD-associated variants in blood, cardiac, and vascular tissues.

SNP (Ref/Alt Allele)	Mark	Blood (Any Cell)	Right Ventricle	Right Atrium	Left Ventricle
rs753856 HSPA6 (C/**G**)	H3K4me1	Enh	-	-	Enh
	H3K4me3	Pro	Pro	-	-
	H3K9ac	Pro	-	-	-
rs706121 BAG1 (T/**C**)	H3K4me1	Enh	-	-	-
	H3K4me3	Pro	-	-	-
	H3K27ac	Enh	-	-	-
rs196329 BAG3 (G/**A**)	H3K4me1	-	Enh	Enh	Enh
	H3K4me3	-	-	-	-
	H3K27ac	Enh	Enh	-	Enh

Table note: H3K4me1—histone H3 lysine 4 mono-methylation; H3K4me3—histone H3 lysine 4 tri-methylation; H3K27ac—acetylation of lysine 27 on histone H3 protein subunit; Enh—histone modification in the enhancer region; Pro—histone modification at the promoter region.

## Data Availability

The original contributions presented in this study are included in the article/supplementary material. Further inquiries can be directed to the corresponding author.

## Supplementary Materials

The following supporting information can be downloaded at https://www.mdpi.com/article/10.3390/ijms27167299/s1.
