# Supplementary material for: Genetic Variants in HSP70 Family and BAG Co-Chaperone Genes: Associations with Coronary Artery Disease Risk and Potential Regulatory Effects
Source: Int J Mol Sci. 2026 Aug 15;27(16):7299. doi: 10.3390/ijms27167299 (PMC13513502; doi:10.3390/ijms27167299)
Supplement: Supplementary file 1 [file ijms-27-07299-s001.zip › ijms-4480814-supplementary.pdf]

Supplementary Table S1. Distribution of SNPs' genotypes in CAD patients/healthy controls and their correspondence to the Hardy-Weinberg equilibrium

SNP	Genotypes	Controls	H_o (H_e)[1]	p[2]	CAD patients	H_o (H_e)[3]	p[4]
rs753856 *HSPA6*	C/C	936 (78.9%)	0.2005 (0.1977)	>0.05	630 (81.6%)	0.1671 (0.2371)	**<0.05**
	C/G	238 (20.1%)			129 (16.7%)		
	G/G	13 (1.1%)			13 (1.7%)		
	Maf(G)	0.111			0.10		
rs13161158 *HSPA4*	T/T	1080 (87.8%)	0.1163 (0.1195)	>0.05	683 (87.6%)	0.1179 (0.1222)	>0.05
	T/C	143 (11.6%)			92 (11.8%)		
	C/C	7 (0.6%)			5 (0.6%)		
	Maf(C)	0.064			0.065		
rs1042665 *HSPA9*	T/T	741 (60.4%)	0.3442 (0.3471)	>0.05	473 (40.2%)	0.3474 (0.3431)	>0.05
	T/C	422 (34.4%)			271 (49.4%)		
	C/C	63 (5.1%)			36 (10.4%)		
	Maf(C)	0.223			0.22		
rs1043618 *HSPA1A*	G/G	505 (41.1%)	0.4553 (0.4618)	>0.05	313 (40.2%)	0.4936 (0.4555)	**<0.05**
	C/G	560 (45.5%)			384 (49.4%)		
	C/C	165 (13.4%)			81 (10.4%)		
	Maf(C)	0.362			0.351		
rs706121 *BAG1*	T/T	856 (74.6%)	0.2232 (0.2438)	**<0.05**	526 (69.9%)	0.2576 (0.2857)	**<0.05**
	T/C	256 (22.3%)			194 (25.8%)		
	C/C	35 (3.1%)			33 (4.4%)		
	Maf(C)	0.142			0.173		
rs17155992 *HSPA14*	G/G	1045 (86.4%)	0.1273 (0.1349)	>0.05	672 (86.2%)	0.1346 (0.1322)	>0.05
	A/G	154 (12.7%)			105 (13.5%)		
	A/A	11 (0.9%)			3 (0.4%)		
	Maf(A)	0.073			0.071		
rs196336 *BAG3*	C/C	402 (33%)	0.484 (0.4897)	>0.05	272 (35%)	0.4801 (0.4838)	>0.05
	C/T	590 (48.4%)			373 (48%)		
	T/T	227 (18.6%)			132 (17%)		
	Maf(T)	0.428			0.41		
rs196329 *BAG3*	G/G	685 (56.2%)	0.3716 (0.3772)	>0.05	457 (58.7%)	0.3586 (0.3577)	>0.05
	A/G	453 (37.2%)			279 (35.9%)		
	A/A	81 (6.6%)			42 (5.4%)		
	Maf(A)	0.252			0.233		
rs1461496 *HSPA8*	G/G	493 (40%)	0.4655 (0.4645)	>0.05	316 (40.6%)	0.4512 (0.4653)	>0.05
	A/G	573 (46.5%)			351 (45.1%)		
	A/A	165 (13.4%)			111 (14.3%)		
	Maf(A)	0.367			0.368		
rs10892958 *HSPA8*	C/C	791 (63.9%)	0.3183 (0.3223)	>0.05	487 (62.6%)	0.3201 (0.3364)	>0.05
	C/G	394 (31.8%)			249 (32%)		
	G/G	53 (4.3%)			42 (5.4%)		
	Maf(G)	0.202			0.214		
rs1136141 *HSPA8*	G/G	889 (75.4%)	0.2248 (0.2315)	>0.05	571 (73.5%)	0.2162 (0.2647)	**<0.05**
	A/G	265 (22.5%)			168 (21.6%)		
	A/A	25 (2.1%)			38 (4.9%)		
	Maf(A)	0.134			0.157		
rs862832 *HSPA12B*	C/C	905 (74%)	0.2371 (0.2429)	>0.05	585 (75.3%)	0.2368 (0.2243)	>0.05
	C/T	290 (23.7%)			184 (23.7%)		
	T/T	28 (2.3%)			8 (1%)		

	Maf(T)	0.141			0.129		
rs910652 *HSPA12B*	T/T	602 (50.3%)	0.4105 (0.413)	>0.05	392 (50.9%)	0.4156 (0.4105)	>0.05
	T/C	491 (41.1%)			320 (41.6%)		
	C/C	103 (8.6%)			58 (7.5%)		
	Maf(C)	0.291			0.283		
[1] – observed (Ho) and expected (He) heterozygosity in healthy controls; [2] – P-HWE in healthy controls; [3] – observed (Ho) and expected (He) heterozygosity in CAD patients; [4] – P-HWE in CAD patients							

Supplementary Table S2. Associations of the analysed SNPs with coronary artery disease risk in the overall study population

SNP	Effect Allele	N	OR [95% CI][1]	*p*-value[2]	p_{perm}[3]
rs753856 *HSPA6*	G	1959	0.89 [0.73-1.1]	0.29	0.33
rs13161158 *HSPA4*	C	2010	1.02 [0.8-1.32]	0.85	0.70
rs1042665 *HSPA9*	C	2006	0.98 [0.84-1.14]	0.79	0.75
rs1043618 *HSPA1A*	C	2008	0.95 [0.83-1.09]	0.48	0.86
rs706121 *BAG1*	C	1900	**1.24 [1.04-1.47]**	**0.015**	**0.019**
rs17155992 *HSPA14*	A	1990	0.98 [0.77-1.25]	0.85	0.86
rs196336 *BAG3*	T	1996	0.93 [0.82-1.05]	0.26	0.25
rs196329 *BAG3*	A	1997	0.9 [0.78-1.05]	0.18	0.20
rs1461496 *HSPA8*	A	2009	1.01 [0.88-1.15]	0.92	0.86
rs10892958 *HSPA8*	G	2016	1.07 [0.92-1.25]	0.36	0.30
rs1136141 *HSPA8*	A	1956	1.19 [1-1.41]	0.05	0.06
rs862832 *HSPA12B*	T	2000	0.9 [0.74-1.08]	0.25	0.39
rs910652 *HSPA12B*	C	1966	0.96 [0.83-1.11]	0.58	0.52
All calculations were performed relative to the minor alleles (Effect allele) with adjustment for sex, age, BMI, smoking; 1 - odds ratio and 95% confidence interval; 2– *p*-value; 3 – *p*-value after the permutation test; statistically significant differences are marked in bold.					

Supplementary Table S3. Stratified associations of the analysed SNPs with coronary artery disease risk according to sex, smoking status, and physical activity level

**SNP**	**Effect Allele**	**N**	**OR [95% CI][1]**	***p*-value[2]**	***p*$_{perm}$[3]**	**N**	**OR [95% CI][1]**	***p*-value[2]**	***p*$_{perm}$[3]**
		Males				***Females***			
rs753856 *HSPA6*	G	1004	0.87 [0.65-1.16]	0.35	0.39	955	0.93 [0.68-1.27]	0.66	0.86
rs13161158 *HSPA4*	C	1034	0.99 [0.7-1.41]	0.97	0.86	976	1.07 [0.73-1.58]	0.73	0.86
rs1042665 *HSPA9*	C	1033	0.91 [0.74-1.12]	0.38	0.86	973	1.06 [0.84-1.34]	0.63	0.86
rs1043618 *HSPA1A*	C	1030	1.02 [0.85-1.23]	0.80	1.00	978	0.94 [0.77-1.16]	0.58	0.43
rs706121 *BAG1*	C	981	**1.48 [1.16-1.88]**	**0.0015**	**0.002**	919	1.07 [0.82-1.39]	0.61	0.86
rs17155992 *HSPA14*	A	1024	0.92 [0.65-1.3]	0.64	0.60	966	1.13 [0.79-1.61]	0.50	0.75
rs196336 *BAG3*	T	1026	0.88 [0.74-1.05]	0.15	0.24	970	0.96 [0.79-1.17]	0.69	0.64
rs196329 *BAG3*	A	1028	**0.82 [0.67-1]**	**0.047**	**0.04**	969	1 [0.79-1.25]	0.97	0.86
rs1461496 *HSPA8*	A	1032	1.1 [0.92-1.31]	0.29	0.33	977	0.86 [0.7-1.05]	0.15	0.12
rs10892958 *HSPA8*	G	1036	1.14 [0.92-1.41]	0.23	0.22	980	0.99 [0.78-1.26]	0.94	1.00
rs1136141 *HSPA8*	A	1016	1.24 [0.98-1.57]	0.07	0.07	940	1.11 [0.85-1.45]	0.46	0.43
rs862832 *HSPA12B*	T	1029	0.86 [0.67-1.1]	0.22	0.23	971	0.88 [0.65-1.19]	0.40	0.50
rs910652 *HSPA12B*	C	1011	1 [0.83-1.22]	0.96	0.86	955	0.88 [0.7-1.1]	0.27	0.31
		Smokers				***Non-smokers***			

rs753856 *HSPA6*	G	748	0.84 [0.6-1.16]	0.28	0.28	1175	0.93 [0.7-1.23]	0.61	0.86
rs13161158 *HSPA4*	C	774	0.87 [0.58-1.28]	0.47	0.48	1197	1.12 [0.79-1.58]	0.52	0.70
rs1042665 *HSPA9*	C	769	0.96 [0.75-1.23]	0.74	0.70	1198	0.99 [0.81-1.21]	0.91	0.86
rs1043618 *HSPA1A*	C	770	0.95 [0.77-1.18]	0.63	0.75	1200	0.96 [0.8-1.16]	0.68	1.00
rs706121 *BAG1*	C	731	**1.39 [1.06-1.82]**	**0.017**	**0.02**	1130	1.13 [0.89-1.43]	0.31	0.36
rs17155992 *HSPA14*	A	760	1.22 [0.82-1.81]	0.32	0.34	1193	0.9 [0.64-1.25]	0.52	0.86
rs196336 *BAG3*	T	769	0.87 [0.7-1.06]	0.17	0.24	1189	0.97 [0.81-1.15]	0.70	0.75
rs196329 *BAG3*	A	769	0.85 [0.67-1.07]	0.16	0.17	1190	0.93 [0.76-1.14]	0.48	0.45
rs1461496 *HSPA8*	A	770	1.02 [0.83-1.25]	0.85	0.75	1201	0.92 [0.76-1.1]	0.36	0.37
rs10892958 *HSPA8*	G	773	1.11 [0.88-1.42]	0.37	0.44	1204	1.06 [0.85-1.31]	0.60	0.70
rs1136141 *HSPA8*	A	761	1.24 [0.94-1.63]	0.13	0.16	1154	1.2 [0.94-1.52]	0.14	0.26
rs862832 *HSPA12B*	T	765	0.9 [0.68-1.2]	0.47	0.46	1196	0.86 [0.66-1.11]	0.25	0.27
rs910652 *HSPA12B*	C	753	1.02 [0.82-1.28]	0.83	0.86	1179	0.9 [0.73-1.09]	0.28	0.34
		Low physical activity				***Normal physical activity***			
rs753856 *HSPA6*	G	901	1.03 [0.78-1.37]	0.81	0.70	665	**0.61 [0.41-0.89]**	0.0098	0.008
rs13161158 *HSPA4*	C	912	1.07 [0.75-1.53]	0.71	0.70	670	0.93 [0.58-1.51]	0.77	0.86
rs1042665 *HSPA9*	C	909	0.92 [0.73-1.15]	0.45	0.36	668	1.11 [0.86-1.45]	0.41	0.34

rs1043618 *HSPA1A*	C	911	0.89 [0.74-1.07]	0.22	0.24	669	0.93 [0.74-1.18]	0.57	0.75
rs706121 *BAG1*	C	873	1.14 [0.89-1.45]	0.30	0.19	644	1.15 [0.86-1.55]	0.35	0.35
rs17155992 *HSPA14*	A	906	0.83 [0.58-1.19]	0.30	0.46	665	1.19 [0.78-1.79]	0.42	0.46
rs196336 *BAG3*	T	908	0.9 [0.74-1.09]	0.27	0.33	670	0.91 [0.74-1.13]	0.42	0.52
rs196329 *BAG3*	A	912	1.01 [0.81-1.25]	0.92	0.86	669	0.78 [0.6-1]	0.05	0.06
rs1461496 *HSPA8*	A	911	1.03 [0.85-1.25]	0.76	0.75	669	1.06 [0.85-1.33]	0.58	0.86
rs10892958 *HSPA8*	G	911	0.97 [0.77-1.22]	0.80	1.00	670	1.23 [0.94-1.59]	0.13	0.14
rs1136141 *HSPA8*	A	887	1.08 [0.84-1.4]	0.53	0.75	649	1.21 [0.91-1.61]	0.20	0.21
rs862832 *HSPA12B*	T	910	0.91 [0.69-1.2]	0.50	0.46	667	1.04 [0.74-1.44]	0.83	0.86
rs910652 *HSPA12B*	C	904	0.96 [0.79-1.18]	0.72	0.70	661	1.09 [0.85-1.39]	0.51	0.75

All calculations were performed relative to the minor alleles (Effect allele); 1 - odds ratio and 95% confidence interval; 2– *p*- value; 3 – *p*-value after the permutation test; statistically significant differences are marked in bold.

Supplementary Table S4. Stratified associations of the analysed SNPs with coronary artery disease risk in males stratified by smoking status

SNP	Effect Allele	N	OR [95% CI][1]	*p*-value[2]	p_{perm}[3]	N	OR [95% CI][1]	*p*-value[2]	p_{perm}[3]
		Smoking males				***Non-smoking males***			
rs753856 *HSPA6*	G	594	0.79 [0.55-1.14]	0.21	0.23	389	0.87 [0.52-1.46]	0.60	0.86
rs13161158 *HSPA4*	C	614	0.89 [0.57-1.39]	0.60	0.67	398	1.18 [0.66-2.11]	0.59	0.70
rs1042665 *HSPA9*	C	611	0.88 [0.66-1.18]	0.39	0.36	400	0.92 [0.66-1.29]	0.65	0.67
rs1043618 *HSPA1A*	C	611	1.01 [0.79-1.28]	0.95	0.86	397	1.03 [0.75-1.41]	0.85	0.86
rs706121 *BAG1*	C	585	**1.36 [1.00-1.85]**	**0.048**	**0.04**	372	**1.53 [0.98-2.40]**	**0.06**	**0.046**
rs17155992 *HSPA14*	A	605	1.04 [0.67-1.61]	0.85	1.00	397	0.69 [0.36-1.34]	0.28	0.29
rs196336 *BAG3*	T	610	0.83 [0.66-1.05]	0.12	0.12	394	0.97 [0.72-1.29]	0.81	0.86
rs196329 *BAG3*	A	610	0.78 [0.60-1.02]	0.07	0.05	396	0.81 [0.58-1.15]	0.25	0.34
rs1461496 *HSPA8*	A	611	1.03 [0.82-1.29]	0.81	0.86	399	1.09 [0.79-1.49]	0.60	0.86
rs10892958 *HSPA8*	G	614	1.16 [0.88-1.52]	0.30	0.28	400	1.20 [0.83-1.72]	0.33	0.29
rs1136141 *HSPA8*	A	608	1.27 [0.93-1.74]	0.13	0.09	384	1.29 [0.87-1.92]	0.20	0.26
rs862832 *HSPA12B*	T	609	0.84 [0.61-1.15]	0.28	0.50	398	0.82 [0.53-1.26]	0.36	0.86
rs910652 *HSPA12B*	C	601	1.06 [0.82-1.35]	0.67	0.75	390	0.91 [0.65-1.27]	0.56	0.60
All calculations were performed relative to the minor alleles (Effect allele); 1 - odds ratio and 95% confidence interval; 2– *p*- value; 3 – *p*-value after the permutation test; statistically significant differences are marked in bold.									

Supplementary Table S5. Analysis of the effect of rs753856 *HSPA6* on the binding of DNA to transcription factors

№	Ref/SNP allele[1]	TF[2]	GAIN /LOSS[3]	Motif[4]	P-Value SNP impact[5]	P-Value Ref[6]	P-Value SNP[7]
1	C/G	HSF2	gain	HSF2_1	0	0.418	2.50E-07
2	C/G	NR3C1	gain	HSF2_1	0	0.418	2.50E-07
3	C/G	HSF1	gain	HSF1_1	0	0.467	0.000001
4	C/G	CUX1	gain	HSF1_1	0	0.467	0.000001
5	C/G	IKZF1	gain	IKZF1_2	0.001	0.180	0.002
6	C/G	RUNX1	gain	IKZF1_2	0.001	0.180	0.002
7	C/G	ZNF263	gain	ZNF263_disc 1	0.000	0.728	0.004

8	C/G	NFKB1	gain	ZNF263_disc1	0.000	0.728	0.004
9	C/G	NFKB	gain	NFKB_5	0.006	0.143	0.006
10	C/G	NFKB	gain	NFKB_1	0.006	0.145	0.006
11	C/G	NFKB1	gain	NFKB1_2	0.003	0.059	0.006
12	C/G	HSF1	gain	NFKB1_2	0.003	0.059	0.006
13	C/G	KLF5	gain	MA0599.1	0.003	0.137	0.008
14	C/G	PAX1	gain	MA0599.1	0.003	0.137	0.008
15	C/G	MAF	gain	MAF_1	0.001	0.101	0.009
16	C/G	HNF1A	gain	MAF_1	0.001	0.101	0.009
17	C/G	BCL	gain	BCL_disc6	0.005	0.111	0.010
18	C/G	SPI1	gain	SPI1_1	0.003	0.236	0.014
19	C/G	RARB	gain	SPI1_1	0.003	0.236	0.014
20	C/G	NFKB	gain	NFKB_2	0.003	0.201	0.015
21	C/G	TP53	gain	NFKB_2	0.003	0.201	0.015
22	C/G	ZNF263	gain	MA0528.1	0.006	0.110	0.015
23	C/G	NFKB	gain	NFKB_3	0.013	0.109	0.015
24	C/G	NFKB	gain	NFKB_disc1	0.003	0.128	0.018
25	C/G	GFI1	gain	NFKB_disc1	0.003	0.128	0.018
26	C/G	PAX4	gain	PAX4_3	0.022	0.180	0.018
27	C/G	NFKB	gain	NFKB_4	0.012	0.147	0.018
28	C/G	SPIC	gain	SPIC_2	0.003	0.473	0.022
29	C/G	CTCF	gain	SPIC_2	0.003	0.473	0.022
30	C/G	CACD	gain	CACD_1	0.005	0.204	0.022
31	C/G	TFAP2	gain	TFAP2_2	0.004	0.292	0.022
32	C/G	RELA	gain	MA0107.1	0.021	0.145	0.023
33	C/G	STAT5A	gain	STAT5A_4	0.005	0.564	0.025
34	C/G	SPDEF	gain	SPDEF_4	0.023	0.168	0.025
35	C/G	CTCFL	gain	CTCFL_disc1	0.018	0.274	0.030
36	C/G	STAT6	gain	STAT6_2	0.017	0.552	0.042
37	C/G	SPI1	gain	SPI1_3	0.015	0.538	0.047
38	C/G	REL	loss	MA0101.1	0.017	0.382	0.050
39	C/G	SPI1	loss	SPI1_2	0.012	0.595	0.050
40	C/G	Mecom	loss	MA0029.1	0.009	0.008	0.053
41	C/G	RUNX1	loss	RUNX1_6	0.003	0.003	0.059
42	C/G	FOXK1	loss	RUNX1_6	0.003	0.003	0.059
43	C/G	HNF4	loss	HNF4_4	0.010	0.010	0.089
44	C/G	TFAP2C	loss	TFAP2C_7	0.006	0.010	0.097
45	C/G	HNF4A	loss	HNF4A_1	0.004	0.008	0.097
46	C/G	RUNX1	loss	RUNX1_8	0.0001	0.007	0.137
47	C/G	GATA	loss	RUNX1_8	0.0001	0.007	0.137
48	C/G	RAD21	loss	RAD21_disc3	0.008	0.014	0.160
49	C/G	NR2F1	loss	MA0017.1	0.023	0.035	0.219
50	C/G	RFX5	loss	MA0510.1	0.003	0.011	0.233
51	C/G	NFATC2	loss	MA0510.1	0.003	0.011	0.233

52	C/G	CBX5	loss	CBX5_1	0.010	0.037	0.262
53	C/G	IRF	loss	IRF_1	0.017	0.026	0.262
54	C/G	RFX3	loss	RFX3_2	0.006	0.028	0.319
55	C/G	SOX13	loss	SOX13_1	0.005	0.025	0.323
56	C/G	RFX1	loss	RFX1_2	0.001	0.013	0.361
57	C/G	HNF4G	loss	RFX1_2	0.001	0.013	0.361
58	C/G	NR4A2	loss	NR4A2_1	0.008	0.021	0.371
59	C/G	RFX2	loss	RFX2_1	0.007	0.046	0.400
60	C/G	HES1	loss	HES1_1	0.013	0.032	0.424
61	C/G	SREBP	loss	SREBP_disc 1	0.005	0.019	0.424
62	C/G	CREB3	loss	CREB3_1	0.008	0.038	0.499
63	C/G	AHR::ARNT	loss	AHR::ARNT _3	0.016	0.041	0.573
64	C/G	FOXG1	loss	FOXG1_2	0.004	0.013	0.825
65	C/G	RXRA	loss	FOXG1_2	0.004	0.013	0.825
66	C/G	GRHL1	loss	GRHL1_2	0.009	0.048	0.896

1 – reference (Ref) / alternative (SNP) allele;
2 – TF - transcription factor;
3 – binding of TF to the reference (LOSS) / alternative (GAIN) allele;
4 – binding sites with high affinity for TF;
5 – p value statistically confirming the potential gain or loss of function of the genomic region with SNP in terms of transcription factor binding;
6 – p-value for assessing the binding of TF to the Ref allele;
7 – p-value for assessing the binding of TF to the SNP allele.

Supplementary Table S6. Analysis of the effect of rs706121 *BAG1* on the binding of DNA to transcription factors.

№	Ref/SNP allele[1]	TF[2]	GAIN /LOSS[3]	Motif[4]	P-Value SNP impact[5]	P-Value Ref[6]	P-Value SNP[7]
1	C/T	SP1	gain	SP1_disc2	0.002	0.087	0.004
2	C/T	ELK1	gain	SP1_disc2	0.002	0.087	0.004
3	C/T	HSFY2	gain	HSFY2_1	0.007	0.062	0.004
4	C/T	Sox2	gain	MA0143.3	0.004	0.085	0.005
5	C/T	ETS	gain	MA0143.3	0.004	0.085	0.005
6	C/T	SRY	gain	SRY_1	0.021	0.084	0.005
7	C/T	FOXA	gain	FOXA_disc5	0.009	0.124	0.011
8	C/T	Sox6	gain	MA0515.1	0.008	0.194	0.015
9	C/T	SOX10	gain	SOX10_2	0.005	0.431	0.016
10	C/T	RUNX1	gain	RUNX1_8	0.003	0.236	0.019
11	C/T	SPIB	gain	RUNX1_8	0.003	0.236	0.019
12	C/T	DMRT2	gain	DMRT2_1	0.011	0.164	0.020
13	C/T	TBP	gain	TBP_4	0.004	0.465	0.025
14	C/T	EP300	gain	TBP_4	0.004	0.465	0.025
15	C/T	FOXP1	gain	MA0481.1	0.007	0.238	0.025
16	C/T	FOXC1	gain	FOXC1_1	0.011	0.209	0.026
17	C/T	Mecom	gain	MA0029.1	0.022	0.224	0.027

18	C/T	SOX4	gain	SOX4_1	0.005	0.425	0.032
19	C/T	SOX11	gain	SOX11_1	0.003	0.431	0.034
20	C/T	PRDM1	gain	SOX11_1	0.003	0.431	0.034
21	C/T	DMRT1	gain	DMRT1_1	0.003	0.255	0.035
22	C/T	PAX1	gain	DMRT1_1	0.003	0.255	0.035
23	C/T	ATF1	gain	ATF1_3	0.019	0.323	0.042
24	C/T	EHF	loss	MA0598.1	0	0.002	0.051
25	C/T	CENPB	loss	MA0598.1	0	0.002	0.051
26	C/T	PAX5	loss	PAX5_disc3	0.022	0.013	0.069
27	C/T	SIX5	loss	SIX5_disc3	0.012	0.014	0.075
28	C/T	ETV2	loss	ETV2_1	0.011	0.007	0.087
29	C/T	ETS	loss	ETS_disc8	0.003	0.003	0.101
30	C/T	FEV	loss	ETS_disc8	0.003	0.003	0.101
31	C/T	STAT2::STAT1	loss	MA0517.1	0.013	0.017	0.106
32	C/T	TEAD1	loss	TEAD1_3	0.016	0.008	0.107
33	C/T	PRDM1	loss	PRDM1_2	0.002	0.014	0.109
34	C/T	AP1	loss	PRDM1_2	0.002	0.014	0.109
35	C/T	ETS	loss	ETS_2	0.006	0.005	0.132
36	C/T	SPIB	loss	MA0081.1	0.011	0.010	0.134
37	C/T	ETS1	loss	ETS1_3	0.018	0.007	0.139
38	C/T	ETS1	loss	ETS1_2	0.001	0.009	0.176
39	C/T	ZNF589	loss	ETS1_2	0.001	0.009	0.176
40	C/T	Ets1	loss	MA0098.2	0.001	0.012	0.193
41	C/T	HNF4G	loss	MA0098.2	0.001	0.012	0.193
42	C/T	ELK1	loss	ELK1_5	0.013	0.013	0.237
43	C/T	ELF1	loss	MA0473.1	0.019	0.021	0.258
44	C/T	SPI1	loss	SPI1_1	0.004	0.029	0.294
45	C/T	ELK1	loss	MA0028.1	0.021	0.030	0.304
46	C/T	FLI1	loss	FLI1_3	0.003	0.011	0.316
47	C/T	RXRA	loss	FLI1_3	0.003	0.011	0.316
48	C/T	ELK1	loss	ELK1_4	0.006	0.010	0.331
49	C/T	SPI1	loss	SPI1_3	0.010	0.020	0.340
50	C/T	ETV4	loss	ETV4_1	0.007	0.017	0.358
51	C/T	GABP	loss	GABP_1	0.010	0.032	0.362
52	C/T	ERG	loss	ERG_3	0.010	0.015	0.407
53	C/T	ERF	loss	ERF_1	0.012	0.017	0.408
54	C/T	AP1	loss	AP1_disc6	0.006	0.029	0.423
55	C/T	POU5F1	loss	POU5F1_disc2	0.019	0.041	0.431
56	C/T	HSF2	loss	HSF2_1	0	0.00000591	0.442
57	C/T	RUNX1	loss	HSF2_1	0	0.00000591	0.442
58	C/T	ETS1	loss	ETS1_4	0.001	0.013	0.456
59	C/T	HNF4A	loss	ETS1_4	0.001	0.013	0.456
60	C/T	ELK4	loss	ELK4_2	0.005	0.016	0.471

61	C/T	HSF1	loss	HSF1_1	0	0.0000020 3	0.476
62	C/T	PBX1	loss	HSF1_1	0	0.0000020 3	0.476
63	C/T	SIX5	loss	SIX5_disc1	0.004	0.019	0.498
64	C/T	FOXO3	loss	SIX5_disc1	0.004	0.019	0.498
65	C/T	ZNF784	loss	ZNF784_1	0.0001	0.003	0.529
66	C/T	SOX10	loss	ZNF784_1	0.0001	0.003	0.529
67	C/T	ETS1	loss	ETS1_6	0.004	0.022	0.786
68	C/T	FEV	loss	ETS1_6	0.004	0.022	0.786

1 – reference (Ref) / alternative (SNP) allele;
2 – TF - transcription factor;
3 – binding of TF to the reference (LOSS) / alternative (GAIN) allele;
4 – binding sites with high affinity for TF;
5 – p value statistically confirming the potential gain or loss of function of the genomic region with SNP in terms of transcription factor binding;
6 – p-value for assessing the binding of TF to the Ref allele;
7 – p-value for assessing the binding of TF to the SNP allele.

Supplementary Table S7. Analysis of the effect of rs196329 *BAG3* on the binding of DNA to transcription factors

№	Ref/SNP allele[1]	TF[2]	GAIN /LOSS[3]	Motif[4]	P-Value SNP impact[5]	P-Value Ref[6]	P-Value SNP[7]
1	A/G	HINFP	gain	HINFP_2	0.0002	0.684	0.001
2	A/G	CTCF	gain	HINFP_2	0.0002	0.684	0.001
3	A/G	Ets1	gain	HINFP_2	0.0002	0.684	0.001
4	A/G	E2F4	gain	MA0470.1	0.0001	0.132	0.002
5	A/G	CTCF	gain	MA0470.1	0.0001	0.132	0.002
6	A/G	MZF1	gain	MA0470.1	0.0001	0.132	0.002
7	A/G	ETV5	gain	ETV5_1	0.004	0.116	0.002
8	A/G	ETV1	gain	ETV5_1	0.004	0.116	0.002
9	A/G	ELF5	gain	ETV5_1	0.004	0.116	0.002
10	A/G	ETV4	gain	ETV4_2	0.003	0.094	0.002
11	A/G	BCL	gain	ETV4_2	0.003	0.094	0.002
12	A/G	TBX20	gain	ETV4_2	0.003	0.094	0.002
13	A/G	ELK1	gain	ELK1_5	0.0003	0.798	0.002
14	A/G	ELF1	gain	ELK1_5	0.0003	0.798	0.002
15	A/G	BHLHE23	gain	ELK1_5	0.0003	0.798	0.002
16	A/G	ELK3	gain	ELK3_2	0.0049	0.092	0.002
17	A/G	E2F1	gain	ELK3_2	0.0049	0.092	0.002
18	A/G	ELK1	gain	ELK1_4	0.0007	0.702	0.002
19	A/G	ETS	gain	ELK1_4	0.0007	0.702	0.002
20	A/G	NKX3-1	gain	ELK1_4	0.0007	0.702	0.002
21	A/G	ETV2	gain	ETV2_1	0.0005	0.199	0.002
22	A/G	GABPA	gain	ETV2_1	0.0005	0.199	0.002
23	A/G	ELF3	gain	ETV2_1	0.0005	0.199	0.002

24	A/G	FEV	gain	FEV_2	0.002	0.170	0.003
25	A/G	ELK1	gain	FEV_2	0.002	0.170	0.003
26	A/G	NFY	gain	FEV_2	0.002	0.170	0.003
27	A/G	NRF1	gain	NRF1_disc2	0.003	0.060	0.003
28	A/G	ERG	gain	NRF1_disc2	0.003	0.060	0.003
29	A/G	RARA	gain	NRF1_disc2	0.003	0.060	0.003
30	A/G	ELK4	gain	ELK4_2	0.001	0.670	0.004
31	A/G	E2F	gain	ELK4_2	0.001	0.670	0.004
32	A/G	NKX3-1	gain	ELK4_2	0.001	0.670	0.004
33	A/G	ERG	gain	ERG_3	0.002	0.809	0.004
34	A/G	ELF1	gain	ERG_3	0.002	0.809	0.004
35	A/G	NR2F6	gain	ERG_3	0.002	0.809	0.004
36	A/G	ELK4	gain	MA0076.2	0.0003	0.087	0.004
37	A/G	MXI1	gain	MA0076.2	0.0003	0.087	0.004
38	A/G	STAT1	gain	MA0076.2	0.0003	0.087	0.004
39	A/G	ETS1	gain	ETS1_4	0.0007	0.514	0.004
40	A/G	DMRTA2	gain	ETS1_4	0.0007	0.514	0.004
41	A/G	NFE2L2	gain	ETS1_4	0.0007	0.514	0.004
42	A/G	FLI1	gain	FLI1_3	0.0009	0.917	0.004
43	A/G	ZNF75A	gain	FLI1_3	0.0009	0.917	0.004
44	A/G	NKX3-2	gain	FLI1_3	0.0009	0.917	0.004
45	A/G	ERF	gain	ERF_1	0.0009	0.427	0.005
46	A/G	ELK3	gain	ERF_1	0.0009	0.427	0.005
47	A/G	EGR1	gain	ERF_1	0.0009	0.427	0.005
48	A/G	SMC3	gain	SMC3_disc1	0.0008	0.621	0.005
49	A/G	RFX5	gain	SMC3_disc1	0.0008	0.621	0.005
50	A/G	RXRA	gain	SMC3_disc1	0.0008	0.621	0.005
51	A/G	SPIC	gain	SPIC_2	0.0007	0.296	0.006
52	A/G	FLI1	gain	SPIC_2	0.0007	0.296	0.006
53	A/G	ELF1	gain	SPIC_2	0.0007	0.296	0.006
54	A/G	HINFP	gain	MA0131.1	0.003	0.116	0.006
55	A/G	Bach1::Mafk	gain	MA0131.1	0.003	0.116	0.006
56	A/G	GATA	gain	MA0131.1	0.003	0.116	0.006
57	A/G	E2F1	gain	MA0024.2	0.002	0.141	0.007
58	A/G	ISL2	gain	MA0024.2	0.002	0.141	0.007
59	A/G	EGR4	gain	MA0024.2	0.002	0.141	0.007
60	A/G	ETS1	gain	ETS1_6	0.003	0.785	0.007
61	A/G	GABPA	gain	ETS1_6	0.003	0.785	0.007
62	A/G	RARG	gain	ETS1_6	0.003	0.785	0.007
63	A/G	ZNF784	gain	ZNF784_1	0.001	0.508	0.009
64	A/G	VDR	gain	ZNF784_1	0.001	0.508	0.009
65	A/G	NFY	gain	ZNF784_1	0.001	0.508	0.009
66	A/G	GABPA	gain	GABPA_2	0.003	0.214	0.009
67	A/G	VDR	gain	GABPA_2	0.003	0.214	0.009
68	A/G	PATZ1	gain	GABPA_2	0.003	0.214	0.009
69	A/G	RARA	gain	RARA_9	0.002	0.297	0.009

70	A/G	SOX17	gain	RARA_9	0.002	0.297	0.009
71	A/G	ELK1	gain	RARA_9	0.002	0.297	0.009
72	A/G	CTCF	gain	CTCF_1	0.002	0.189	0.011
73	A/G	EGR3	gain	CTCF_1	0.002	0.189	0.011
74	A/G	SPIB	gain	CTCF_1	0.002	0.189	0.011
75	A/G	SIN3A	gain	SIN3A_disc5	0.002	0.167	0.012
76	A/G	E2F3	gain	SIN3A_disc5	0.002	0.167	0.012
77	A/G	ZNF354C	gain	SIN3A_disc5	0.002	0.167	0.012
78	A/G	RAD21	gain	RAD21_disc 1	0.001	0.439	0.013
79	A/G	MZF1	gain	RAD21_disc 1	0.001	0.439	0.013
80	A/G	EP300	gain	RAD21_disc 1	0.001	0.439	0.013
81	A/G	PTF1A	gain	PTF1A_1	0.002	0.120	0.013
82	A/G	EGR2	gain	PTF1A_1	0.002	0.120	0.013
83	A/G	ELK1	gain	PTF1A_1	0.002	0.120	0.013
84	A/G	ELF4	gain	ELF4_1	0.002	0.641	0.014
85	A/G	NR2F1	gain	ELF4_1	0.002	0.641	0.014
86	A/G	ELF5	gain	ELF4_1	0.002	0.641	0.014
87	A/G	RXRA	gain	RXRA_disc2	0.001	0.444	0.014
88	A/G	RARA	gain	RXRA_disc2	0.001	0.444	0.014
89	A/G	RARB	gain	RXRA_disc2	0.001	0.444	0.014
90	A/G	RORA	gain	RORA_6	0.002	0.309	0.014
91	A/G	SPI1	gain	RORA_6	0.002	0.309	0.014
92	A/G	RXRG	gain	RORA_6	0.002	0.309	0.014
93	A/G	EGR1	gain	EGR1_disc2	0.002	0.220	0.015
94	A/G	RXRB	gain	EGR1_disc2	0.002	0.220	0.015
95	A/G	TGIF1	gain	EGR1_disc2	0.002	0.220	0.015
96	A/G	YY1	gain	YY1_disc5	0.004	0.384	0.017
97	A/G	E2F1	gain	YY1_disc5	0.004	0.384	0.017
98	A/G	TATA	gain	YY1_disc5	0.004	0.384	0.017
99	A/G	E2F1	gain	E2F1_18	0.004	0.522	0.018
100	A/G	GATA	gain	E2F1_18	0.004	0.522	0.018
101	A/G	SPI1	gain	E2F1_18	0.004	0.522	0.018
102	A/G	E2F1	gain	E2F1_9	0.004	0.426	0.019
103	A/G	RXRB	gain	E2F1_9	0.004	0.426	0.019
104	A/G	RXRA	gain	E2F1_9	0.004	0.426	0.019
105	A/G	E2F1	gain	E2F1_15	0.005	0.478	0.021
106	A/G	NKX2-3	gain	E2F1_15	0.005	0.478	0.021
107	A/G	HNF1B	gain	E2F1_15	0.005	0.478	0.021
108	A/G	PAX5	gain	PAX5_disc3	0.005	0.277	0.021
109	A/G	ZNF589	gain	PAX5_disc3	0.005	0.277	0.021
110	A/G	Atoh1	gain	PAX5_disc3	0.005	0.277	0.021
111	A/G	EHF	gain	EHF_1	0.005	0.505	0.029
112	A/G	RARA	gain	EHF_1	0.005	0.505	0.029
113	A/G	ELF3	gain	ELF3_3	0.004	0.773	0.031

114	A/G	HNF4	gain	ELF3_3	0.004	0.773	0.031
115	A/G	STAT3	gain	ELF3_3	0.004	0.773	0.031
116	A/G	TEAD1	loss	TEAD1_2	0.001	0.003	0.053
117	A/G	PROP1	loss	TEAD1_2	0.001	0.003	0.053
118	A/G	HDAC2	loss	TEAD1_2	0.001	0.003	0.053
119	A/G	Nkx3-2	loss	MA0122.1	0.002	0.001	0.085
120	A/G	ETS	loss	MA0122.1	0.002	0.001	0.085
121	A/G	NR2C2	loss	MA0122.1	0.002	0.001	0.085
122	A/G	NKX3-2	loss	NKX3-2_1	0.001	0.001	0.140
123	A/G	NFE2L2	loss	NKX3-2_1	0.001	0.001	0.140
124	A/G	NKX2-8	loss	NKX3-2_1	0.001	0.001	0.140
125	A/G	PPARG	loss	PPARG_1	0.001	0.011	0.141
126	A/G	EGR3	loss	PPARG_1	0.001	0.011	0.141
127	A/G	TATA	loss	PPARG_1	0.001	0.011	0.141
128	A/G	Nkx2-5 (var.2)	loss	MA0503.1	0.002	0.006	0.173
129	A/G	EHF	loss	MA0503.1	0.002	0.006	0.173
130	A/G	HNF1A	loss	MA0503.1	0.002	0.006	0.173
131	A/G	VDR	loss	VDR_1	0	0	0.210
132	A/G	RXRA::VDR	loss	VDR_1	0	0	0.210
133	A/G	Arnt::Ahr	loss	VDR_1	0	0	0.210
134	A/G	EGR2	loss	EGR2_1	0.004	0.010	0.226
135	A/G	NEUROD2	loss	EGR2_1	0.004	0.010	0.226
136	A/G	CREB1	loss	EGR2_1	0.004	0.010	0.226
137	A/G	SOX8	loss	SOX8_6	0.001	0.009	0.253
138	A/G	FLI1	loss	SOX8_6	0.001	0.009	0.253
139	A/G	NR2F6	loss	SOX8_6	0.001	0.009	0.253
140	A/G	EGR1	loss	EGR1_5	0.001	0.007	0.433
141	A/G	FOXO1	loss	EGR1_5	0.001	0.007	0.433
142	A/G	ELF5	loss	EGR1_5	0.001	0.007	0.433
143	A/G	NKX3-2	loss	NKX3-2_2	0.003	0.019	0.461
144	A/G	EGR2	loss	NKX3-2_2	0.003	0.019	0.461
145	A/G	BATF	loss	NKX3-2_2	0.003	0.019	0.461
146	A/G	RARG	loss	RARG_1	0.005	0.039	0.625
147	A/G	MEIS2	loss	RARG_1	0.005	0.039	0.625

1 – reference (Ref) / alternative (SNP) allele;
2 – TF - transcription factor;
3 – binding of TF to the reference (LOSS) / alternative (GAIN) allele;
4 – binding sites with high affinity for TF;
5 – p value statistically confirming the potential gain or loss of function of the genomic region with SNP in terms of transcription factor binding;
6 – p-value for assessing the binding of TF to the Ref allele;
7 – p-value for assessing the binding of TF to the SNP allele.

Supplementary Table S8. Baseline and clinical characteristics of the studied groups.

Baseline and clinical characteristics	IHD patients (n=836)	Controls (n=1328)	P-value

Age, Me [Q1; Q3]		62 [57; 67]	59 [54; 67]	**<0.001**
Gender	Males, N(%)	530 (81.8%)	567 (28.7%)	**<0.05**
	Females, N(%)	304 (18.2%)	761 (71.3%)	
Smoking	Yes, N (%)	416 (75.1%)	404 (34.8%)	**<0.05**
	No, N (%)	412 (24.9%)	923 (65.2%)	
Low fruit/vegetable consumption	Yes, N (%)	325 (38.9%)	351 (26.4%)	>0.05
	No, N (%)	471 (56.4%)	515 (38.8%)	
	ND, N (%)	40 (4.8%)	462 (34.8%)	
Body mass index, Me [Q1; Q3]		29 [26; 33] (n=773)	27.1 [26; 29.3] (n=874)	**<0.05**
Family history of IHD	Yes, N (%)	446 (53.4%)	0	
	No, N (%)	22 (2.6%)	0	
	ND, N (%)	368 (44%)	1328 (100%)	
Age at onset of IHD, Me [Q1; Q3]		55 [49; 61] (n=755)	-	
Age at onset of hypertension, Me [Q1; Q3]		50 [43; 56] (n=750)	-	
Myocardial infarction	Yes, N (%)	584 (84.9%)	-	
	No, N (%)	252 (15.1%)	-	
Platelets, Me [Q1; Q3]		252.5 [207; 300] (n=254)	ND	
Total cholesterol, mmol/L, Me [Q1; Q3]		5.39 [4.6; 6.6] (n=747)	ND	
Triglycerides, mmol/L, Me [Q1; Q3]		1.36 [0.98; 2.02] (n=715)	ND	
Glucose level, mmol/L, Me [Q1; Q3]		5.03 [4.41; 6.1] (n=790)	ND	
LDL, Me [Q1; Q3]		3.99 [3.1; 4.63] (n=533)	ND	
HDL, Me [Q1; Q3]		1.3 [1.02; 1.5] (n=553)	ND	
International normalized ratio, Me [Q1; Q3]		1.07 [1; 1.25] (n=281)	ND	
Activated partial thromboplastin time, seconds, Me [Q1; Q3]		29 [25.9; 36.9] (n=279)	ND	
Activated plasma recalcification time (APRT), seconds, Me [Q1; Q3]		121 [105; 135] (n=173)	ND	
Prothrombin index, Me [Q1; Q3]		94 [83; 100] (n=319)	ND	
Fibrinogen A, g/L, Me [Q1; Q3]		4.5 [3.7; 5.22] (n=268)	ND	
Total fibrinogen, g/L, Me [Q1; Q3]		4.5 [3.7; 5.18] (n=195)	ND	
Coronary angiography	Yes, N (%)	247	-	
	No, N (%)	16	-	
Atherosclerosis of the LCA (left coronary artery)	Yes, N (%)	124	-	
	No, N (%)	122	-	
Stenosis of the LCA	Less than 50%, N (%)	101	-	

	More than 50%, N (%)	23	-	
Stenosis of the LCA, %, Me [Q1; Q3]		45 [35; 60] (n=44)	-	
Atherosclerosis of the RCA (right coronary artery)	Yes, N (%)	208	-	
	No, N (%)	38	-	
Stenosis of the RCA	Less than 50%, N (%)	102	-	
	More than 50%, N (%)	106	-	
Stenosis of the RCA, %, Me [Q1; Q3]		80 [50; 99] (n=143)	-	
Atherosclerosis of the circumflex artery	Yes, N (%)	188	-	
	No, N (%)	59	-	
Stenosis of the circumflex artery	Less than 50%, N (%)	121	-	
	More than 50%, N (%)	66	-	
Stenosis of the circumflex artery, %, Me [Q1; Q3]		65 [50; 90] (n=119)		-
Atherosclerosis of the anterior interventricular artery (AIV)	Yes, N (%)	210	-	
	No, N (%)	30	-	
Stenosis of the AIV	Less than 50%, N (%)	91	-	
	More than 50%, N (%)	119	-	
Stenosis of the AIV, %, Me [Q1; Q3]		70 [50; 98.5] (n=160)	-	
Statistically significant differences between groups are indicated in bold; ND – no data.				

Supplementary Table S9. Regulatory annotation of *HSP70* and *BAG* variants using HaploReg v4.2 and RegulomeDB

SNP	EUR freq	HaploReg V4.2 (1)								RegulomeDB (2)		atSNP (3)
		Promoter histone marks	Enhancer histone marks	DNAse	Proteins bound	Motifs changed	GRASP QTL hits	Selected eQTL hits	dbSNP func annot	probability	ranking	TF binding
rs1043618 *HSPA1A*	0.39			49 tissues	10 bound proteins		1 hit	37 hits	5'-UTR	0.554	1f	+
rs13161158 *HSPA4*	0.08		7 tissues	SKIN		CTCF,Rad21,SMC3		2 hits	intronic	0.708	1f	+
rs753856 *HSPA6*	0.12	BLD	FAT			Evi-1,Ik-2		2 hits	missense	0.554	1f	+
rs1042665 *HSPA9*	0.25			HRT, SKIN		Brachyury, Eomes		1 hit	synonymous	0.554	1f	+
rs17155992 *HSPA14*	0.06					GR,Pax-5,p300		2 hits	intronic	0.553	1f	+
rs706121 *BAG1*	0.83		LNG, BLD	CRVX, BLD, SKIN	JUND, MAFK		1 hit	30 hits	intronic	0.554	1f	+
rs196336 *BAG3*	0.65	14 tissues	18 tissues	22 tissues	5 bound proteins	5 altered motifs		6 hits	intronic	0.403	1b	+
rs196329 *BAG3*	0.8		9 tissues			Ets,Nkx3	1 hit	5 hits	intronic	0.554	1f	+
rs1461496 *HSPA8*	0.63	HRT	13 tissues	6 tissues			1 hit		intronic	0.482	2b	+
rs10892958 *HSPA8*	0.17	N/D	N/D	N/D	N/D	N/D	N/D	N/D	N/D	0.554	1f	+
rs1136141 *HSPA8*	0.14	N/D	N/D	N/D	N/D	N/D	N/D	N/D	N/D	0.554	1f	+
rs862832 *HSPA12B*	0.14	SKIN	17 tissues	4 tissues		Irf,Maf	1 hit	3 hits	intronic	0.554	1f	+
rs910652 *HSPA12B*	0.33		GI, LIV	MUS, LNG, LIV			2 hits	3 hits	intronic	0.195	1f	+

1 –analysis of regulatory potential SNPs using the HaploReg V4.2 resource (https://pubs.broadinstitute.org/mammals/haploreg/haploreg.php): analysed Promoter histone marks, Enhancer histone marks; regions of hypersensitivity to DNase-1 (DNAse), bounding proteins; Motifs changed; GRASP QTL hits; Selected eQTL hits (expression or molecular QTL variants linked to trait-associated haplotype blocks); 2 – analysis of regulatory potential SNPs using the RegulomeDB resource (https://regulomedb.org/regulome-summary/): regulatory coefficients are presented - 1b (eQTL/caQTL + TF binding + any motif + Footprint + chromatin accessibility peak), 2b (TF binding + any motif + Footprint + chromatin accessibility peak), 1f (eQTL/caQTL + TF binding / chromatin accessibility peak); 3 - analysis of transcription factors binding using the atSNP resource (http://atsnp.biostat.wisc.edu/search): The effect of SNPs on DNA binding to transcription factors is presented (+/-); N/D – no data available

Supplementary Table S10. Primer and probe sequences, annealing temperatures, and Mg^{2+} concentrations for the analysed SNPs

SNP	Primers and probes	Annealing temperature, °C	$MgCl_2$ concentration, mM
rs753856 *HSPA6*	F: 5′-TGAGGATGAGGCCCAGAGG-3′<br>R: 5′-AGCCAGGCAAGGACTTCC-3′<br>5′-(FAM)CCTTAGGGA**C**AAGATTCC(RTQ1)-3′<br>5′-(ROX)CCTTAGGGA**G**AAGATTCC(BHQ2)-3′	58.0	2.5
rs13161158 *HSPA4*	F: 5′- CCAGTGGCTGATACACCAGA-3′<br>R: 5′-GGAAGACACTTACGCATTCCA-3′<br>5′-(FAM)CCATCTAG**T**GCCCCTAGCT(RTQ1)-3′<br>5′-(ROX) CCATCTAG**C**GCCCCTAGCT(BHQ2)-3′	62.0	3.0
rs1042665 *HSPA9*	F: 5′-AAATAAGCTCCGGCTGAAAA-3′<br>R: 5′-GACAGGGGTTGATTTGACTAAA-3′<br>5′-(FAM)AGTTCACA**T**TTAGCCT(RTQ1)-3′<br>5′-(ROX)AGTTCACA**C**TTAGCCT(BHQ2)-3′	51.0	2.5
rs1043618 *HSPA1A*	F: 5′-ATCCAGTGTTCCGTTTCCAG-3′<br>R: 5′-GAGTAGGTGGTGCCCAGGT-3′<br>5′-(FAM)CTCAGAGC**G**GAGCCGAC(RTQ1)-3′<br>5′-(ROX)CTCAGAGC**C**GAGCCGAC(BHQ2)-3′	62.5	2.5
rs706121 *BAG1*	F: 5′- CAGCCCTCTCTCATCACCTT -3′<br>R: 5′- TTCCAGTTCAGGAGCCTCTC -3′<br>5′-(FAM)-CTGAACCTT**C**CTTTCCAGTC-(RTQ1)-3′<br>5′-(ROX)-CTGAACCTT**T**CTTTCCAGTC-(BHQ2)-3′	61.0	2.0
rs17155992 *HSPA14*	F: 5′-TCCTTCAGCCCTGTAATGATGT-3′<br>R: 5′-GGCTTCAGTATCAACCATCTTCT-3′<br>5′-(FAM)CTAACATTATGAC**G**TTGTGTCTTC(RTQ1)-3′<br>5′-(ROX)CTAACATTATGAC**A**TTGTGTCTTC(BHQ2)-3′	58.0	3.0
rs196336 *BAG3*	F: 5′- CACCTGCAGTCTTCCTGGAT -3′<br>R: 5′- CATCCGGCCTCTGTTTATTC -3′	60.0	3.0

	5′-(FAM)CAGGCCC**T**TTCCTGGC(RTQ1)-3′<br>5′-(ROX)CAGGCCC**C**TTCCTGGC(BHQ2)-3′		
rs196329 *BAG3*	F: 5′-AGTGTCCACCACACCCTTTC-3′<br>R: 5′-GGTTCAGCATGGGCAAAG-3′<br>5′-(FAM)TCACCTTCC**A**CTCAGTTAC(RTQ1)-3′<br>5′-(ROX)TCACCTTCC**G**CTCAGTTAC(BHQ2)-3′	59.0	3.0
rs1461496 *HSPA8*	F: 5′- CTCGCCTTCATAAACCTTGG -3′<br>R: 5′- TGGATTCCAAAACCATTCGT -3′<br>5′-(FAM)TCGCTCAAACATCCA(RTQ1)-3′<br>5′-(ROX)TCGCTCAGACATCCA(BHQ2)-3′	51.0	3.0
rs10892958 *HSPA8*	F: 5′- CCTTGGACATGGTTGCTGAA -3′<br>R: 5′- CGTGCTTAGACAGGAGGTGA-3′<br>5′-(FAM)CCAGGAAAAACGTATGGCCACTGC(RTQ1)-3′<br>5′-(ROX)CCAGGAAAAAGGTATGGCCACTGC(BHQ2)-3′	65.0	4.5
rs1136141 *HSPA8*	F: 5′- AGAACCCCCAAGCCTCAG -3′<br>R: 5′- GGAAACCGGTCTCATTGAACT -3′<br>5′-(FAM)TACCTGGG**G**TGTAGGCC(RTQ1)-3′<br>5′-(ROX)TACCTGGG**A**TGTAGGCC(BHQ2)-3′	61.5	3.0
rs862832 *HSPA12B*	F: 5′-TGTGGTGGTGGCAGCTAC-3′<br>R: 5′-CAGCATTTCAGGGCAGGAC-3′<br>5′-(FAM)CAGGGCTACTC**C**ACTCCCTCC(RTQ1)-3′<br>5′-(ROX)CAGGGCTACTC**T**ACTCCCTCC(BHQ2)-3′	65.0	2.5
rs910652 *HSPA12B*	F: 5′-ATCTCAAGCCTGCCTGTGG-3′<br>R: 5′-CCGTCCTGATCTTTGCACAG-3′<br>5′-(FAM)CTACACTT**T**TCACTGGAAG(RTQ1)-3′<br>5′-(ROX)CTACACTT**C**TCACTGGAAG(BHQ2)-3′	58.0	4.0
